# Supplementary material for: Donor Pericardial Interleukin and Apolipoprotein Levels May Predict the Outcome after Human Orthotopic Heart Transplantation
Source: Int J Mol Sci. 2023 Apr 5;24(7):6780. doi: 10.3390/ijms24076780 (PMC10095178; doi:10.3390/ijms24076780)
Supplement: Supplementary file 1 [file ijms-24-06780-s001.zip › ijms-2236543-supplementary.pdf]

**SUPPLEMENTARY TABLES**

**Table S1. Relationship between donor risk scores and immune parameters**

| | UNOS-D score | HDS | aHDS | Median | IQR (25-75) |
|---|---|---|---|---|---|
| | p value | p value | p value | | |
| thiol (414 nm) | 0.284 | 0.174 | 0.362 | 0.73 | 0.00 - 3.05 |
| thiol (405 nm) | 0.541 | 0.520 | 0.353 | 1.32 | 0.00 - 3.66 |
| IFNg (pg/mL) | 0.896 | 0.935 | 0.970 | 2.74 | 1.72 - 3.56 |
| IL-17A (pg/mL) | 0.004 | 0.000 | 0.009 | 8.56 | 8.49 - 8.72 |
| IL-17F (pg/mL) | 0.405 | 0.396 | 0.928 | 17.56 | 17.20 - 18.41 |
| IL-21 (pg/mL) | 0.722 | 0.582 | 0.840 | 27.46 | 19.20 - 37.94 |
| IL-22 (pg/mL) | 0.722 | 0.654 | 0.862 | 17.11 | 17.09 - 17.13 |
| IL-4 (pg/mL) | 0.748 | 0.973 | 0.998 | 25.06 | 20.72 - 32.65 |
| TNF (pg/mL) | 0.374 | 0.427 | 0.568 | 3.22 | 3.14 - 3.48 |
| IL-10 (pg/mL) | 0.748 | 0.365 | 0.450 | 25.90 | 6.10 - 42.40 |
| IL-13 (pg/mL) | 0.426 | 0.733 | 0.982 | 5.83 | 5.69 - 6.40 |
| IL-2 (pg/mL) | 0.549 | 0.518 | 0.287 | 22.81 | 14.78 - 34.51 |
| IL-5 (pg/mL) | 0.004 | 0.000 | 0.000 | 1.04 | 0.93 - 1.31 |
| IL-6 (pg/mL) | 0.469 | 0.053 | 0.057 | 111.32 | 31.99 - 390.71 |
| IL-9 (pg/mL) | 0.818 | 0.840 | 0.494 | 17.39 | 16.83 - 18.75 |
| Leptin (ng/mL) | 0.014 | 0.653 | 0.061 | 5.14 | 4.58 - 5.39 |
| Adiponectin (ng/mL) | 0.006 | 0.030 | 0.142 | 16.07 | 12.18 - 23.55 |
| Adipsin (ng/mL) | 0.963 | 0.303 | 0.553 | 530.14 | 469.19 - 603.05 |
| oxLDL (ng/mL) | 0.499 | 0.573 | 0.202 | 242.28 | 170.83 - 289.91 |
| ApoAI (ug/mL) | 0.416 | 0.972 | 0.005 | 51.85 | 41.52 - 94.00 |
| ApoAII (ug/mL) | 0.109 | 0.973 | 0.030 | 194.83 | 108.36 - 230.30 |
| ApoB100 (ug/mL) | 0.691 | 0.881 | 0.228 | 105.72 | 50.51 - 219.23 |
| ApoCII (ug/mL) | 0.329 | 0.568 | 0.022 | 9.28 | 8.40 - 10.06 |
| ApoCIII (ug/mL) | 0.357 | 0.541 | 0.059 | 5.77 | 1.84 - 17.88 |
| ApoD (ug/mL) | 0.350 | 0.774 | 0.098 | 6.87 | 4.07 - 11.52 |
| ApoH (ug/mL) | 0.744 | 0.783 | | 0.00 | 0.00 - 0.00 |
| ApoJ (ug/mL) | 0.860 | 0.426 | | 0.00 | 0.00 - 0.00 |
| ApoM (ug/mL) | 0.607 | 0.823 | 0.053 | 11.35 | 2.90 - 28.98 |
| T3 (pg/mL) | 0.121 | 0.756 | 0.312 | 2.00 | 1.10 - 2.62 |
| T4 (ug/dL) | 0.262 | 0.002 | 0.438 | 3.26 | 2.84 - 3.78 |

*aHDS: adapted Heart Donor Score; Apo: apolipoprotein; HDS: Heart Donor Score; IFNg: interferon-γ; IL: interleukin; IQR: interquartile range; oxLDL: oxidized low-density lipoprotein; T3: triiodothyronine; T4: thyroxine; TNF: tumor necrosis factor; UNOS-D: United Network for Organ Sharing Donor score*

**Table S2. Relationship between donor interleukin levels and primary graft dysfunction**

| | NO PGD | | PGD | | p value |
|---|---|---|---|---|---|
| | Median | IQR (25-75) | Median | IQR (25-75) | |
| thiol (414 nm) | 0.80 | 0.00 - 3.18 | 0.65 | 0.00 - 6.56 | 0.964 |
| thiol (405 nm) | 1.60 | 0.00 - 3.69 | 0.52 | 0.00 - 6.22 | 0.688 |
| IFNg (pg/mL) | 2.30 | 1.51 - 5.94 | 3.23 | 2.60 - 3.47 | 0.238 |
| IL-17A (pg/mL) | 8.59 | 8.49 - 8.83 | 8.54 | 8.51 - 8.63 | 0.485 |
| IL-17F (pg/mL) | 17.56 | 17.17 - 24.15 | 17.83 | 17.21 - 18.24 | 0.965 |
| IL-21 (pg/mL) | 32.25 | 20.39 - 38.72 | 19.74 | 17.31 - 53.94 | 0.206 |
| IL-22 (pg/mL) | 17.11 | 17.08 - 17.13 | 17.13 | 17.09 - 17.19 | 0.590 |
| IL-4 (pg/mL) | 25.93 | 20.72 - 44.16 | 20.72 | 18.11 - 25.93 | 0.148 |
| TNF (pg/mL) | 3.21 | 3.07 - 3.60 | 3.30 | 3.19 - 3.40 | 0.631 |
| IL-10 (pg/mL) | 32.50 | 6.10 - 45.70 | 19.30 | 6.10 - 45.70 | 0.591 |
| IL-13 (pg/mL) | 5.76 | 5.68 - 6.93 | 5.91 | 5.76 - 6.19 | 0.727 |
| IL-2 (pg/mL) | 20.13 | 13.44 - 40.20 | 24.14 | 18.79 - 30.16 | 0.662 |
| IL-5 (pg/mL) | 1.05 | 0.94 - 1.40 | 1.02 | 0.89 - 1.14 | 0.221 |
| IL-9 (pg/mL) | 17.45 | 16.90 - 18.90 | 16.90 | 16.77 - 179.06 | 0.359 |
| Leptin (ng/mL) | 5.17 | 4.43 - 5.37 | 4.83 | 4.72 - 8.88 | 0.359 |
| Adiponectin (ng/mL) | 16.45 | 12.13 - 25.57 | 13.10 | 10.86 - 20.18 | 0.359 |
| Adipsin (ng/mL) | 541.85 | 468.63 - 603.61 | 522.54 | 461.78 - 582.13 | 0.631 |
| oxLDL (ng/mL) | 242.28 | 185.12 - 308.96 | 194.65 | 123.20 - 261.33 | 0.203 |
| ApoAI (ug/mL) | 57.23 | 43.96 - 99.21 | 44.87 | 22.74 - 84.12 | 0.407 |
| ApoAII (ug/mL) | 206.93 | 113.07 - 254.22 | 163.54 | 74.88 - 204.34 | 0.206 |
| ApoB100 (ug/mL) | 106.45 | 52.09 - 231.25 | 65.01 | 48.49 - 182.38 | 0.570 |
| ApoCII (ug/mL) | 9.41 | 8.42 - 16.42 | 8.90 | 8.22 - 9.42 | 0.176 |
| ApoCIII (ug/mL) | 7.84 | 3.32 - 33.35 | 2.30 | 1.56 - 7.04 | 0.176 |
| ApoD (ug/mL) | 6.94 | 3.98 - 19.22 | 5.56 | 4.05 - 9.69 | 0.570 |
| ApoH (ug/mL) | 0.00 | 0.00 - 0.00 | 0.00 | 0.00 - 0.00 | 0.292 |
| ApoJ (ug/mL) | 0.00 | 0.00 - 0.00 | 0.00 | 0.00 - 0.00 | 0.564 |
| ApoM (ug/mL) | 17.91 | 7.01 - 34.07 | 8.58 | 0.00 - 11.35 | 0.105 |
| T3 (pg/mL) | 2.00 | 0.53 - 2.83 | 2.06 | 1.32 - 2.49 | 0.920 |
| T4 (ug/dL) | 2.95 | 2.57 - 3.78 | 3.52 | 3.25 - 6.36 | 0.194 |

*Apo: apolipoprotein; IFNg: interferon-γ; IL: interleukin; IQR: interquartile range; oxLDL: oxidized low-density lipoprotein; PGD: primer graft dysfunction; T3: triiodothyronine; T4: thyroxine; TNF: tumor necrosis factor*

**Table S3. Relationship between donor interleukin levels and postoperative mechanical circulatory support**

| | NO MCS | | MCS | | p value |
|---|---|---|---|---|---|
| | Median | IQR (25-75) | Median | IQR (25-75) | |
| thiol (414 nm) | 0.40 | 0.00 - 3.30 | 1.38 | 0.00 - 4.77 | 0.665 |
| thiol (405 nm) | 1.32 | 0.00 - 3.82 | 1.31 | 0.00 - 4.67 | 0.933 |
| IFNg (pg/mL) | 2.51 | 1.51 - 6.18 | 3.12 | 2.08 - 3.37 | 0.509 |
| IL-17A (pg/mL) | 8.63 | 8.50 - 8.86 | 8.54 | 8.46 - 8.61 | 0.173 |
| IL-17F (pg/mL) | 17.52 | 17.10 - 24.47 | 17.69 | 17.28 - 18.14 | 0.967 |
| IL-21 (pg/mL) | 33.56 | 20.05 - 44.56 | 20.33 | 17.44 - 40.45 | 0.187 |
| IL-22 (pg/mL) | 17.11 | 17.08 - 17.14 | 17.12 | 17.10 - 17.16 | 0.734 |
| IL-4 (pg/mL) | 26.79 | 21.59 - 44.16 | 20.72 | 19.42 - 25.06 | 0.082 |
| TNF (pg/mL) | 3.22 | 3.11 - 3.61 | 3.26 | 3.14 - 3.37 | 0.804 |
| IL-10 (pg/mL) | 25.90 | 6.10 - 45.70 | 25.90 | 6.10 - 39.10 | 0.767 |
| IL-13 (pg/mL) | 5.79 | 5.66 - 7.41 | 5.88 | 5.70 - 6.06 | 0.967 |
| IL-2 (pg/mL) | 21.47 | 14.44 - 40.87 | 23.47 | 14.44 - 27.82 | 0.869 |
| IL-5 (pg/mL) | 1.16 | 0.96 - 1.45 | 0.97 | 0.87 - 1.09 | 0.069 |
| IL-9 (pg/mL) | 17.54 | 16.87 - 26.70 | 16.90 | 16.79 - 98.12 | 0.248 |
| Leptin (ng/mL) | 5.14 | 4.37 - 5.38 | 5.05 | 4.75 - 7.39 | 0.248 |
| Adiponectin (ng/mL) | 16.07 | 11.65 - 26.02 | 15.13 | 11.58 - 19.78 | 0.458 |
| Adipsin (ng/mL) | 536.49 | 454.35 - 601.93 | 525.85 | 466.32 - 638.93 | 0.805 |
| oxLDL (ng/mL) | 256.57 | 180.36 - 318.49 | 194.65 | 139.87 - 251.81 | 0.147 |
| ApoAI (ug/mL) | 58.75 | 43.15 - 104.11 | 51.05 | 25.07 - 78.47 | 0.458 |
| ApoAII (ug/mL) | 200.16 | 111.50 - 260.50 | 179.91 | 88.21 - 222.86 | 0.509 |
| ApoB100 (ug/mL) | 112.35 | 43.23 - 263.17 | 74.44 | 49.24 - 144.53 | 0.509 |
| ApoCII (ug/mL) | 9.41 | 8.38 - 16.56 | 9.03 | 8.31 - 9.76 | 0.364 |
| ApoCIII (ug/mL) | 9.02 | 2.91 - 41.21 | 4.18 | 1.59 - 7.88 | 0.248 |
| ApoD (ug/mL) | 6.87 | 3.91 - 19.38 | 6.48 | 4.20 - 9.11 | 0.741 |
| ApoH (ug/mL) | 0.00 | 0.00 - 0.05 | 0.00 | 0.00 - 0.00 | 0.232 |
| ApoJ (ug/mL) | 0.00 | 0.00 - 0.00 | 0.00 | 0.00 - 0.00 | 0.513 |
| ApoM (ug/mL) | 17.70 | 5.64 - 34.49 | 9.64 | 0.00 - 16.52 | 0.282 |
| T3 (pg/mL) | 2.02 | 0.48 - 2.88 | 1.99 | 1.48 - 2.37 | 0.853 |
| T4 (ug/dL) | 2.91 | 2.48 - 3.79 | 3.69 | 3.28 - 5.51 | 0.096 |

*Apo: apolipoprotein; IFNg: interferon-γ; IL: interleukin; IQR: interquartile range; MCS: mechanical circulatory support; oxLDL: oxidized low-density lipoprotein; T3: triiodothyronine; T4: thyroxine; TNF: tumor necrosis factor*

**Table S4. Relationship between donor interleukin levels and vasoplegia**

| | NO vasoplegia | | Vasoplegia | | p value |
|---|---|---|---|---|---|
| | Median | IQR (25-75) | Median | IQR (25-75) | |
| thiol (414 nm) | 0.00 | 0.00 - 2.40 | 4.79 | 2.11 - 11.01 | 0.030 |
| thiol (405 nm) | 0.73 | 0.00 - 3.17 | 6.54 | 2.10 - 10.35 | 0.045 |
| IFNg (pg/mL) | 2.30 | 1.60 - 4.79 | 3.00 | 2.77 - 3.28 | 0.427 |
| IL-17A (pg/mL) | 8.59 | 8.49 - 8.78 | 8.54 | 8.53 - 8.55 | 0.525 |
| IL-17F (pg/mL) | 17.56 | 17.32 - 21.23 | 17.17 | 17.08 - 18.44 | 0.491 |
| IL-21 (pg/mL) | 32.25 | 19.71 - 50.40 | 19.74 | 19.00 - 20.92 | 0.153 |
| IL-22 (pg/mL) | 17.11 | 17.09 - 17.15 | 17.11 | 17.08 - 17.13 | 0.513 |
| IL-4 (pg/mL) | 24.19 | 20.72 - 38.95 | 25.93 | 15.51 - 27.66 | 0.559 |
| TNF (pg/mL) | 3.23 | 3.10 - 3.54 | 3.21 | 3.21 - 3.45 | 0.791 |
| IL-10 (pg/mL) | 32.50 | 12.70 - 45.70 | 6.10 | 6.10 - 19.30 | 0.082 |
| IL-13 (pg/mL) | 5.82 | 5.69 - 6.58 | 5.85 | 5.68 - 6.45 | 1.000 |
| IL-2 (pg/mL) | 24.14 | 14.11 - 37.52 | 20.13 | 14.78 - 22.81 | 0.524 |
| IL-5 (pg/mL) | 1.05 | 0.94 - 1.36 | 1.01 | 0.87 - 1.02 | 0.204 |
| IL-6 (pg/mL) | 181.36 | 35.81 - 421.91 | 36.72 | 29.97 - 51.52 | 0.153 |
| IL-9 (pg/mL) | 17.45 | 16.86 - 34.49 | 16.90 | 16.75 - 17.17 | 0.185 |
| Leptin (ng/mL) | 5.17 | 4.60 - 5.38 | 4.78 | 4.20 - 5.91 | 0.874 |
| Adiponectin (ng/mL) | 16.45 | 12.22 - 24.65 | 13.10 | 10.13 - 19.38 | 0.427 |
| Adipsin (ng/mL) | 541.85 | 474.57 - 615.26 | 470.87 | 411.49 - 522.54 | 0.153 |
| oxLDL (ng/mL) | 242.28 | 161.30 - 299.44 | 280.39 | 194.65 - 289.91 | 0.559 |
| ApoAI (ug/mL) | 46.48 | 42.34 - 92.59 | 72.82 | 27.41 - 168.65 | 0.634 |
| ApoAII (ug/mL) | 196.27 | 109.93 - 226.30 | 163.54 | 48.21 - 339.08 | 0.711 |
| ApoB100 (ug/mL) | 106.45 | 51.04 - 207.22 | 65.01 | 47.00 - 1242.80 | 0.958 |
| ApoCII (ug/mL) | 9.41 | 8.34 - 10.02 | 9.15 | 8.39 - 16.42 | 0.958 |
| ApoCIII (ug/mL) | 6.07 | 2.50 - 16.97 | 2.30 | 1.49 - 33.35 | 0.560 |
| ApoD (ug/mL) | 6.81 | 3.86 - 9.32 | 11.99 | 4.35 - 24.98 | 0.315 |
| ApoH (ug/mL) | 0.00 | 0.00 - 0.00 | 0.00 | 0.00 - 17.15 | 0.349 |
| ApoJ (ug/mL) | 0.00 | 0.00 - 0.00 | 0.00 | 0.00 - 7.35 | 0.017 |
| ApoM (ug/mL) | 11.99 | 4.27 - 27.84 | 8.58 | 0.00 - 132.20 | 0.832 |
| T3 (pg/mL) | 1.99 | 0.82 - 2.72 | 2.23 | 1.59 - 2.50 | 0.506 |
| T4 (ug/dL) | 3.26 | 2.84 - 3.73 | 4.67 | 1.57 - 6.19 | 0.894 |

*Apo: apolipoprotein; IFNg: interferon-γ; IL: interleukin; IQR: interquartile range; oxLDL: oxidized low-density lipoprotein; T3: triiodothyronine; T4: thyroxine; TNF: tumor necrosis factor*

**Table S5. Relationship between donor interleukin levels and rejection**

| | NO rejection | | Rejection | | p value |
|---|---|---|---|---|---|
| | Median | IQR (25-75) | Median | IQR (25-75) | |
| thiol (414 nm) | 0.65 | 0.00 - 3.18 | 1.69 | 0.00 - 3.74 | 0.819 |
| thiol (405 nm) | 1.03 | 0.00 - 3.69 | 1.60 | 0.00 - 4.66 | 0.894 |
| IFNg (pg/mL) | 2.81 | 1.51 - 3.65 | 2.72 | 2.00 - 5.31 | 0.896 |
| IL-17A (pg/mL) | 8.58 | 8.49 - 8.73 | 8.55 | 8.46 - 8.76 | 0.694 |
| IL-17F (pg/mL) | 17.56 | 17.08 - 18.44 | 17.56 | 17.32 - 21.67 | 0.827 |
| IL-21 (pg/mL) | 29.64 | 19.74 - 38.72 | 24.94 | 17.98 - 92.99 | 0.631 |
| IL-22 (pg/mL) | 17.11 | 17.11 - 17.13 | 17.11 | 17.08 - 17.20 | 0.719 |
| IL-4 (pg/mL) | 24.19 | 18.98 - 33.74 | 25.93 | 21.59 - 39.81 | 0.540 |
| TNF (pg/mL) | 3.23 | 3.13 - 3.49 | 3.21 | 3.13 - 3.51 | 0.861 |
| IL-10 (pg/mL) | 19.30 | 6.10 - 32.50 | 32.50 | 12.70 - 45.70 | 0.622 |
| IL-13 (pg/mL) | 5.85 | 5.68 - 6.45 | 5.76 | 5.69 - 7.43 | 0.896 |
| IL-2 (pg/mL) | 22.81 | 14.78 - 34.85 | 22.81 | 16.78 - 44.21 | 0.694 |
| IL-5 (pg/mL) | 1.05 | 0.93 - 1.32 | 1.01 | 0.90 - 1.32 | 0.570 |
| IL-6 (pg/mL) | 102.68 | 36.72 - 389.06 | 119.95 | 24.08 - 537.55 | 0.861 |
| IL-9 (pg/mL) | 17.42 | 16.90 - 18.90 | 16.90 | 16.66 - 734.86 | 0.570 |
| Leptin (ng/mL) | 5.12 | 4.64 - 5.40 | 5.17 | 4.31 - 5.44 | 0.827 |
| Adiponectin (ng/mL) | 16.45 | 12.40 - 23.73 | 12.13 | 10.18 - 21.37 | 0.315 |
| Adipsin (ng/mL) | 522.54 | 468.63 - 601.37 | 576.17 | 476.67 - 658.01 | 0.176 |
| oxLDL (ng/mL) | 242.28 | 166.07 - 289.91 | 194.65 | 166.07 - 299.44 | 0.759 |
| ApoAI (ug/mL) | 45.48 | 27.41 - 83.50 | 118.81 | 50.60 - 234.40 | 0.061 |
| ApoCII (ug/mL) | 9.13 | 8.26 - 9.70 | 10.09 | 9.19 - 21.83 | 0.061 |
| ApoCIII (ug/mL) | 5.21 | 1.62 - 12.56 | 18.80 | 5.79 - 57.93 | 0.074 |
| ApoD (ug/mL) | 5.57 | 3.98 - 10.13 | 8.51 | 5.93 - 26.05 | 0.150 |
| ApoH (ug/mL) | 0.00 | 0.00 - 0.00 | 0.00 | 0.00 - 21.94 | 0.058 |
| ApoJ (ug/mL) | 0.00 | 0.00 - 0.00 | 0.00 | 0.00 - 3.67 | 0.083 |
| T3 (pg/mL) | 2.01 | 0.96 - 2.67 | 2.00 | 1.10 - 2.88 | 1.000 |
| T4 (ug/dL) | 3.23 | 2.77 - 3.83 | 3.66 | 2.47 - 3.99 | 0.853 |

*Apo: apolipoprotein; IFNg: interferon-γ; IL: interleukin; IQR: interquartile range; oxLDL: oxidized low-density lipoprotein; T3: triiodothyronine; T4: thyroxine; TNF: tumor necrosis factor*

**Table S6. Effects of desmopressin replacement therapy on interleukin levels**

| | NO desmopressin therapy | | Desmopressin therapy | | p value |
|---|---|---|---|---|---|
| | Median | IQR (25-75) | Median | IQR (25-75) | |
| thiol (414 nm) | 1.17 | 0.00 - 3.30 | 0.40 | 0.00 - 3.05 | 0.808 |
| thiol (405 nm) | 1.32 | 0.13 - 3.32 | 1.41 | 0.00 - 4.06 | 0.937 |
| IFNg (pg/mL) | 3.44 | 1.92 - 7.61 | 2.46 | 1.56 - 2.95 | 0.143 |
| IL-17A (pg/mL) | 8.68 | 8.52 - 8.88 | 8.54 | 8.48 - 8.64 | 0.143 |
| IL-17F (pg/mL) | 17.93 | 17.47 - 26.14 | 17.45 | 17.10 - 18.21 | 0.189 |
| IL-21 (pg/mL) | 33.56 | 22.70 - 82.31 | 22.93 | 19.01 - 35.45 | 0.165 |
| IL-22 (pg/mL) | 17.13 | 17.09 - 17.23 | 17.11 | 17.09 - 17.13 | 0.249 |
| IL-4 (pg/mL) | 28.53 | 21.59 - 48.71 | 23.32 | 19.42 - 27.23 | 0.141 |
| TNF (pg/mL) | 3.33 | 3.20 - 3.70 | 3.19 | 3.08 - 3.41 | 0.122 |
| IL-10 (pg/mL) | 39.10 | 9.40 - 55.60 | 19.30 | 6.10 - 32.50 | 0.251 |
| IL-13 (pg/mL) | 5.92 | 5.76 - 9.46 | 5.77 | 5.68 - 6.17 | 0.217 |
| IL-2 (pg/mL) | 29.50 | 17.79 - 52.91 | 17.45 | 13.77 - 27.49 | 0.075 |
| IL-5 (pg/mL) | 1.26 | 0.97 - 1.54 | 1.02 | 0.88 - 1.22 | 0.142 |
| IL-6 (pg/mL) | 182.51 | 49.48 - 610.69 | 77.10 | 30.08 - 369.45 | 0.375 |
| IL-9 (pg/mL) | 17.85 | 17.02 - 270.57 | 17.04 | 16.79 - 17.72 | 0.164 |
| Leptin (ng/mL) | 5.28 | 4.91 - 5.65 | 4.72 | 4.25 - 5.26 | 0.054 |
| Adiponectin (ng/mL) | 18.72 | 12.20 - 26.91 | 14.99 | 10.74 - 22.12 | 0.487 |
| oxLDL (ng/mL) | 213.70 | 106.53 - 292.29 | 256.57 | 194.65 - 289.91 | 0.261 |
| ApoAI (ug/mL) | 45.18 | 30.96 - 78.95 | 71.92 | 41.74 - 98.26 | 0.396 |
| ApoAII (ug/mL) | 194.88 | 102.86 - 218.29 | 194.83 | 125.69 - 249.24 | 0.877 |
| ApoB100 (ug/mL) | 105.72 | 50.61 - 251.37 | 95.28 | 48.27 - 183.18 | 0.787 |
| ApoCII (ug/mL) | 9.01 | 8.15 - 18.49 | 9.55 | 8.58 - 10.06 | 0.355 |
| ApoCIII (ug/mL) | 5.34 | 2.15 - 53.67 | 6.95 | 1.84 - 17.88 | 1.000 |
| ApoD (ug/mL) | 5.50 | 3.71 - 16.64 | 7.78 | 4.65 - 11.52 | 0.396 |
| ApoH (ug/mL) | 0.00 | 0.00 - 0.16 | 0.00 | 0.00 - 0.00 | 0.321 |
| ApoJ (ug/mL) | 0.00 | 0.00 - 0.00 | 0.00 | 0.00 - 0.00 | 0.414 |
| ApoM (ug/mL) | 9.63 | 1.75 - 31.20 | 14.31 | 3.29 - 28.98 | 0.699 |
| T3 (pg/mL) | 2.00 | 0.52 - 2.45 | 2.12 | 1.80 - 2.83 | 0.433 |
| T4 (ug/dL) | 3.28 | 2.64 - 3.60 | 3.26 | 2.84 - 4.21 | 0.563 |

*Apo: apolipoprotein; IFNg: interferon-γ; IL: interleukin; IQR: interquartile range; oxLDL: oxidized low-density lipoprotein; T3: triiodothyronine; T4: thyroxine; TNF: tumor necrosis factor*

**Table S7. Effects of glucocorticoid replacement therapy on interleukin levels**

| | NO glucocorticoid therapy | | Glucocorticoid therapy | | p value |
|---|---|---|---|---|---|
| | Median | IQR (25-75) | Median | IQR (25-75) | |
| thiol (414 nm) | 0.32 | 0.00 - 3.29 | 1.46 | 0.00 - 3.05 | 0.686 |
| thiol (405 nm) | 0.78 | 0.00 - 3.29 | 2.49 | 0.18 - 4.06 | 0.431 |
| IFNg (pg/mL) | 2.51 | 1.80 - 3.27 | 2.79 | 1.56 - 7.37 | 0.589 |
| IL-17A (pg/mL) | 8.54 | 8.49 - 8.65 | 8.69 | 8.44 - 8.91 | 0.354 |
| IL-17F (pg/mL) | 17.52 | 17.14 - 18.01 | 17.93 | 17.20 - 25.10 | 0.418 |
| IL-21 (pg/mL) | 30.94 | 18.11 - 37.79 | 25.11 | 19.36 - 55.46 | 0.817 |
| IL-22 (pg/mL) | 17.11 | 17.08 - 17.13 | 17.12 | 17.11 - 17.18 | 0.404 |
| IL-4 (pg/mL) | 23.32 | 19.42 - 27.66 | 29.83 | 21.59 - 48.71 | 0.163 |
| TNF (pg/mL) | 3.22 | 3.14 - 3.34 | 3.35 | 3.09 - 3.63 | 0.562 |
| IL-10 (pg/mL) | 19.30 | 6.10 - 42.40 | 32.50 | 19.30 - 42.40 | 0.342 |
| IL-13 (pg/mL) | 5.83 | 5.70 - 5.99 | 5.98 | 5.69 - 8.37 | 0.616 |
| IL-2 (pg/mL) | 22.81 | 14.78 - 27.49 | 26.82 | 13.77 - 42.21 | 0.643 |
| IL-5 (pg/mL) | 1.03 | 0.93 - 1.21 | 1.27 | 0.90 - 1.52 | 0.463 |
| IL-6 (pg/mL) | 127.82 | 24.75 - 371.09 | 111.32 | 33.11 - 610.14 | 0.643 |
| IL-9 (pg/mL) | 17.39 | 16.83 - 18.75 | 17.17 | 16.81 - 42.01 | 1.000 |
| Adiponectin (ng/mL) | 17.91 | 12.18 - 26.45 | 14.99 | 10.70 - 21.56 | 0.487 |
| Adipsin (ng/mL) | 555.32 | 509.02 - 603.05 | 474.57 | 380.25 - 620.56 | 0.217 |
| oxLDL (ng/mL) | 242.28 | 166.07 - 301.82 | 218.46 | 170.83 - 289.91 | 0.816 |
| ApoAI (ug/mL) | 59.65 | 31.55 - 94.00 | 51.36 | 41.74 - 144.24 | 1.000 |
| ApoAII (ug/mL) | 185.37 | 102.86 - 216.82 | 212.61 | 129.07 - 317.86 | 0.355 |
| ApoB100 (ug/mL) | 106.57 | 53.74 - 219.23 | 68.17 | 25.52 - 977.89 | 0.728 |
| ApoCII (ug/mL) | 9.14 | 8.40 - 10.04 | 9.41 | 8.47 - 14.81 | 0.643 |
| ApoCIII (ug/mL) | 5.77 | 1.79 - 17.24 | 6.53 | 2.10 - 28.80 | 0.817 |
| ApoD (ug/mL) | 6.48 | 3.90 - 11.52 | 6.87 | 4.37 - 20.77 | 0.758 |
| ApoH (ug/mL) | 0.00 | 0.00 - 0.00 | 0.00 | 0.00 - 12.86 | 0.264 |
| ApoJ (ug/mL) | 0.00 | 0.00 - 0.00 | 0.00 | 0.00 - 0.00 | 0.221 |
| ApoM (ug/mL) | 9.64 | 1.75 - 23.65 | 18.27 | 3.76 - 106.68 | 0.296 |
| T3 (pg/mL) | 1.99 | 0.33 - 2.62 | 2.17 | 1.89 - 2.77 | 0.364 |
| T4 (ug/dL) | 3.21 | 2.85 - 3.69 | 3.46 | 2.37 - 4.14 | 0.804 |

*Apo: apolipoprotein; IFNg: interferon-γ; IL: interleukin; IQR: interquartile range; oxLDL: oxidized low-density lipoprotein; T3: triiodothyronine; T4: thyroxine; TNF: tumor necrosis factor*

**Table S8. Effects of L-Thyroxine replacement therapy on interleukin levels**

| | NO L-thyroxine therapy | | L-thyroxine therapy | | p value |
|---|---|---|---|---|---|
| | Median | IQR (25-75) | Median | IQR (25-75) | |
| thiol (414 nm) | 0.32 | 0.00 - 3.30 | 1.46 | 0.00 - 3.21 | 0.665 |
| thiol (405 nm) | 0.78 | 0.00 - 3.59 | 2.49 | 0.54 - 4.80 | 0.312 |
| IFNg (pg/mL) | 2.51 | 1.72 - 3.37 | 2.79 | 1.65 - 7.52 | 0.650 |
| IL-17A (pg/mL) | 8.54 | 8.49 - 8.68 | 8.69 | 8.51 - 8.94 | 0.248 |
| IL-17F (pg/mL) | 17.52 | 17.03 - 18.14 | 17.93 | 17.27 - 24.77 | 0.386 |
| IL-21 (pg/mL) | 30.94 | 19.20 - 46.13 | 22.67 | 19.02 - 42.22 | 0.621 |
| IL-22 (pg/mL) | 17.11 | 17.08 - 17.14 | 17.12 | 17.10 - 17.15 | 0.799 |
| IL-4 (pg/mL) | 24.19 | 20.28 - 28.10 | 29.83 | 20.07 - 54.57 | 0.385 |
| TNF (pg/mL) | 3.22 | 3.11 - 3.37 | 3.35 | 3.14 - 3.61 | 0.620 |
| IL-10 (pg/mL) | 25.90 | 6.10 - 45.70 | 25.90 | 16.00 - 72.10 | 0.767 |
| IL-13 (pg/mL) | 5.83 | 5.66 - 6.12 | 5.98 | 5.70 - 7.93 | 0.592 |
| IL-2 (pg/mL) | 22.81 | 14.44 - 29.83 | 26.82 | 14.44 - 40.87 | 0.649 |
| IL-5 (pg/mL) | 1.03 | 0.93 - 1.29 | 1.27 | 0.97 - 1.51 | 0.343 |
| IL-6 (pg/mL) | 142.02 | 32.73 - 406.58 | 80.58 | 30.30 - 542.25 | 0.934 |
| IL-9 (pg/mL) | 17.43 | 16.88 - 29.03 | 16.90 | 16.77 - 25.83 | 0.364 |
| Leptin (ng/mL) | 5.19 | 4.75 - 5.62 | 4.60 | 4.19 - 5.16 | 0.058 |
| Adiponectin (ng/mL) | 17.91 | 12.26 - 26.02 | 14.05 | 10.04 - 18.63 | 0.248 |
| Adipsin (ng/mL) | 555.32 | 496.11 - 609.43 | 474.57 | 395.40 - 560.94 | 0.161 |
| oxLDL (ng/mL) | 242.28 | 163.69 - 287.53 | 266.10 | 158.92 - 339.93 | 0.562 |
| ApoAI (ug/mL) | 71.92 | 39.82 - 96.36 | 45.15 | 33.44 - 85.09 | 0.364 |
| ApoAII (ug/mL) | 194.83 | 105.48 - 222.30 | 197.68 | 86.26 - 275.43 | 0.934 |
| ApoB100 (ug/mL) | 112.47 | 61.25 - 237.96 | 52.28 | 12.49 - 373.60 | 0.149 |
| ApoCII (ug/mL) | 9.28 | 8.41 - 11.81 | 9.26 | 8.21 - 11.57 | 0.741 |
| ApoCIII (ug/mL) | 7.04 | 2.13 - 30.29 | 4.27 | 1.48 - 14.22 | 0.364 |
| ApoD (ug/mL) | 7.10 | 4.20 - 13.80 | 6.25 | 3.72 - 12.36 | 0.680 |
| ApoH (ug/mL) | 0.00 | 0.00 - 0.00 | 0.00 | 0.00 - 4.29 | 0.894 |
| ApoJ (ug/mL) | 0.00 | 0.00 - 0.00 | 0.00 | 0.00 - 1.84 | 0.127 |
| ApoM (ug/mL) | 11.35 | 5.26 - 27.69 | 13.96 | 1.15 - 55.64 | 0.967 |
| T4 (ug/dL) | 3.21 | 2.71 - 3.67 | 3.52 | 2.66 - 4.46 | 0.539 |

*Apo: apolipoprotein; IFNg: interferon-γ; IL: interleukin; IQR: interquartile range; oxLDL: oxidized low-density lipoprotein; T3: triiodothyronine; T4: thyroxine; TNF: tumor necrosis factor*

**Table S9. Correlations with preexplantation laboratory parameters (1)**

| | INR | | | RBC (T/L) | | | HCT (%) | | |
|---|---|---|---|---|---|---|---|---|---|
| | ρ | p value | N | ρ | p value | N | ρ | p value | N |
| IL-22 (pg/mL) | .485* | 0.030 | 20 | -.555* | 0.026 | 16 | -.525* | 0.030 | 17 |
| IL-10 (pg/mL) | | | | -.583* | 0.018 | 16 | -.532* | 0.028 | 17 |
| IL-21 (pg/mL) | | | | -.504* | 0.046 | 16 | -.483* | 0.050 | 17 |
| IL-9 (pg/mL) | | | | -.545* | 0.029 | 16 | -.519* | 0.033 | 17 |
| Adipsin (ng/mL) | | | | | | | -.486* | 0.048 | 17 |

| | ALT (UI/L) | | | AST (UI/L) | | |
|---|---|---|---|---|---|---|
| | ρ | p value | N | ρ | p value | N |
| IL-22 (pg/mL) | .578** | 0.010 | 19 | .521* | 0.022 | 19 |
| IL-10 (pg/mL) | .513* | 0.025 | 19 | .587** | 0.008 | 19 |
| IL-13 (pg/mL) | .461* | 0.047 | 19 | | | |
| IL-2 (pg/mL) | .542* | 0.017 | 19 | | | |
| IFNg (pg/mL) | .478* | 0.038 | 19 | | | |

| | HGB (g/L) | | |
|---|---|---|---|
| | ρ | p value | N |
| IL-17F (pg/mL) | .467* | 0.038 | 20 |
| IL-4 (pg/mL) | .593** | 0.006 | 20 |
| ApoAII (ug/mL) | .456* | 0.043 | 20 |

| | ALP (UI/L) | | |
|---|---|---|---|
| | ρ | p value | N |
| ApoAII (ug/mL) | -.588* | 0.013 | 17 |
| ApoM (ug/mL) | -.572* | 0.016 | 17 |

*ALP: alkaline phosphatase; ALT: alanine transaminase; Apo: apolipoprotein; AST: aspartate aminotransferase; HCT: hematocrit; HGB: hemoglobin; IFN: : interferon-γ; IL: interleukin; INR: international normalized ratio. RBC: red blood cell. *: p < 0.05; **: p < 0.01.*

**Table S10. Correlations with preexplantation laboratory parameters (2)**

| | CRP (mg/L) | | |
|---|---|---|---|
| | ρ | p value | N |
| Leptin (ng/mL) | -.517* | 0.020 | 20 |

| | CKMB (UI/L) | | |
|---|---|---|---|
| | ρ | p value | N |
| oxLDL (ng/mL) | -.540* | 0.025 | 17 |

| | PLT (G/L) | | |
|---|---|---|---|
| | ρ | p value | N |
| T3 (pg/mL) | .501* | 0.040 | 17 |

| | Creatinine (µmol/L) | | |
|---|---|---|---|
| | ρ | p value | N |
| thiol (414 nm) | -.494* | 0.027 | 20 |

| | Sodium (mmol/L) | | |
|---|---|---|---|
| | ρ | p value | N |
| Adiponectin (ng/mL) | .466* | 0.044 | 19 |

| | Potassium (mmol/L) | | |
|---|---|---|---|
| | ρ | p value | N |
| ApoB100 (ug/mL) | .525* | 0.021 | 19 |
| ApoH (ug/mL) | .472* | 0.041 | 19 |

*Apo: apolipoprotein; CKMB: creatin kinase-MB isoform CRP: C-reactive protein; oxLDL: oxidized low-density lipoprotein; PLT: platelet count; T3: triiodothyronine. *: $p < 0.05$; **: $p < 0.01$.*

**Table S11. Correlations with preexplantation echocardiography parameters**

| TAPSE (cm) | | |
|---|---|---|
| IL-5 (pg/mL) | ρ | .975** |
| | p value | 0.005 |
| | N | 5 |
| ApoAI (ug/mL) | ρ | -.975** |
| | p value | 0.005 |
| | N | 5 |
| ApoB100 (ug/mL) | ρ | -.947* |
| | p value | 0.014 |
| | N | 5 |
| ApoM (ug/mL) | ρ | -.947* |
| | p value | 0.014 |
| | N | 5 |

| Left Atrial Horizontal Diameter (cm) | | |
|---|---|---|
| ApoAII (ug/mL) | ρ | -.644* |
| | p value | 0.044 |
| | N | 10 |
| ApoCII (ug/mL) | ρ | -.718* |
| | p value | 0.019 |
| | N | 10 |
| ApoM (ug/mL) | ρ | -.702* |
| | p value | 0.024 |
| | N | 10 |

| Posterior Wall Diastolic Diameter (cm) | | |
|---|---|---|
| ApoAII (ug/mL) | ρ | -.593* |
| | p value | 0.033 |
| | N | 13 |

| Right Ventricular Diastolic Diameter (cm) | | |
|---|---|---|
| IFNg (pg/mL) | ρ | -.735* |
| | p value | 0.038 |
| | N | 8 |
| IL-17F (pg/mL) | ρ | -.766* |
| | p value | 0.027 |
| | N | 8 |

| Septum Diastolic Diameter (cm) | | |
|---|---|---|
| TNF (pg/mL) | ρ | -.591* |
| | p value | 0.043 |
| | N | 12 |
| IL-13 (pg/mL) | ρ | -.597* |
| | p value | 0.040 |
| | N | 12 |

| LVESD diameter (cm) | | |
|---|---|---|
| T4 (ug/dL) | ρ | -.556* |
| | p value | 0.021 |
| | N | 17 |

| Left Atrial Longitudinal Diameter (cm) | | |
|---|---|---|
| thiol (414 nm) | ρ | .750* |
| | p value | 0.013 |
| | N | 10 |

*Apo: apolipoprotein; IFNg: interferon-γ; IL: interleukin; LVESD: left ventricular end-systolic diameter; T4: thyroxine TAPSE: tricuspid annular plane systolic excursion; TNF: tumor necrosis factor. *: $p < 0.05$; **: $p < 0.01$.*

**Table S12. Correlation between donor age and immune parameters**

| | ρ | p |
|---|---|---|
| thiol (414 nm) | 0.192 | 0.417 |
| thiol (405 nm) | 0.229 | 0.331 |
| IFNg (pg/mL) | -0.059 | 0.806 |
| IL-17A (pg/mL) | 0.003 | 0.989 |
| IL-17F (pg/mL) | -0.261 | 0.266 |
| IL-21 (pg/mL) | 0.067 | 0.778 |
| IL-22 (pg/mL) | -0.010 | 0.966 |
| IL-4 (pg/mL) | -,467* | 0.038 |
| TNF (pg/mL) | -0.073 | 0.759 |
| IL-10 (pg/mL) | -0.055 | 0.819 |
| IL-13 (pg/mL) | -0.114 | 0.633 |
| IL-2 (pg/mL) | -0.226 | 0.338 |
| IL-5 (pg/mL) | 0.038 | 0.874 |
| IL-6 (pg/mL) | -0.057 | 0.812 |
| IL-9 (pg/mL) | 0.052 | 0.827 |
| Leptin (ng/mL) | 0.408 | 0.074 |
| Adiponectin (ng/mL) | 0.282 | 0.229 |
| Adipsin (ng/mL) | -0.017 | 0.945 |
| oxLDL (ng/mL) | -0.089 | 0.709 |
| ApoAI (ug/mL) | -,595** | 0.006 |
| ApoAII (ug/mL) | -,613** | 0.004 |
| ApoB100 (ug/mL) | -0.409 | 0.073 |
| ApoCII (ug/mL) | -,525* | 0.018 |
| ApoCIII (ug/mL) | -,470* | 0.037 |
| ApoD (ug/mL) | -,504* | 0.023 |
| ApoH (ug/mL) | -0.367 | 0.112 |
| ApoJ (ug/mL) | -0.380 | 0.099 |
| ApoM (ug/mL) | -,674** | 0.001 |
| T3 (pg/mL) | -0.055 | 0.824 |
| T4 (ug/dL) | 0.151 | 0.538 |

*Apo: apolipoprotein; IFNg: interferon-γ; IL: interleukin; oxLDL: oxidized low-density lipoprotein; T3: triiodothyronine; T4: thyroxine; TNF: tumor necrosis factor.* *: $p < 0.05$; **: $p < 0.01$.
